# Effects of levodopa/carbidopa intestinal gel versus oral levodopa/carbidopa on B vitamin levels and neuropathy

**DOI:** 10.1002/brb3.698

**Published:** 2017-04-07

**Authors:** Sebastian Loens, Elena Chorbadzhieva, Alexandra Kleimann, Dirk Dressler, Christoph Schrader

**Affiliations:** ^1^ Department of Neurology and Clinical NeurophysiologyHannover Medical SchoolHannoverGermany; ^2^ Department of PsychiatrySocial Psychiatry and PsychotherapyHannover Medical SchoolHannoverGermany

**Keywords:** carbidopa, cobalamin, folate, levodopa, neuropathy, Parkinson′s disease, pyridoxine

## Abstract

**Objectives:**

To determine the possible interactions between levodopa therapy and plasma levels of B vitamins in patients with advanced idiopathic Parkinson's disease (IPD) in the context of either oral levodopa therapy or levodopa/carbidopa intestinal gel (LCIG). Secondly, to determine the prevalence of neuropathy and its relation to plasma levels of B vitamins and homocysteine.

**Methods:**

Medication doses, neurographies, and serum levels of pyridoxine, cobalamin, folate, and homocysteine of eight LCIG and 13 orally treated advanced IPD patients matched for age, Hoehn & Yahr stage, and UPRDS III were collected. This data was analyzed for correlation with daily levodopa dose (LDD).

**Results:**

LICG patients had a longer disease duration and higher LDD. All LCIG patients and most orally treated patients had sensorimotor axonal polyneuropathy. Of all plasma vitamin levels, pyridoxine was decreased most and significantly lower in the LCIG group. Cobalamin and folate, however, were within the lower reference range, and homocysteine highly elevated, all without any significant difference between both groups. LDD correlated significantly with pyridoxine deficiency (*p* = .02) irrespective of the route of application and with hyperhomocysteinemia in the LCIG group (*p* = .03). At LDDs above 2,000 mg, pyridoxine deficiency was almost always detectable.

**Conclusions:**

Pyridoxine deficiency and hyperhomocysteinemia are dependent on the daily levodopa/carbidopa dose, while levels of cobalamin and folate are not. The mode of application of levodopa/carbidopa has no impact on B‐vitamin levels. Neuropathy is very frequent in advanced IPD; however, it remains to be investigated further whether neuropathy is more frequent in LCIG than in orally levodopa/carbidopa‐treated advanced IPD patients.

## 1 Introduction

Since more than 40 years, levodopa is the most effective treatment for idiopathic Parkinson's disease (IPD), but due to its short plasma half‐life it causes pulsatile striatal receptor stimulation, and thereby—on the long run—motor side effects such as dyskinesias and motor fluctuations (Olanow, Obeso, & Stocchi, 2006). Continuous jejunal infusion of levodopa/carbidopa intestinal gel (LCIG) via a percutaneous pump leads to more constant plasma levels of levodopa (Nyholm et al., 2003), and it results in significant reduction in off‐time and on‐time with troublesome dyskinesias (Antonini et al., 2007; Eggert et al., 2008; Fernandez et al., 2013; Olanow et al., 2014). Common side effects of LCIG treatment are surgical‐ or device‐related complications (Olanow et al., 2014), but there are also anecdotal reports on neuropathy in LCIG‐treated patients.

Currently, two types of neuropathy during LCIG treatment have been described: Slowly progressive neuropathy which can also be observed in advanced IPD, and subacute neuropathy, sometimes worsening within days reminding of non‐disease‐related autoimmune neuropathies like Guillain–Barré syndrome. It remains unclear if these two types are different in nature or just mark opposite ends of a spectrum.

**Figure 1:**
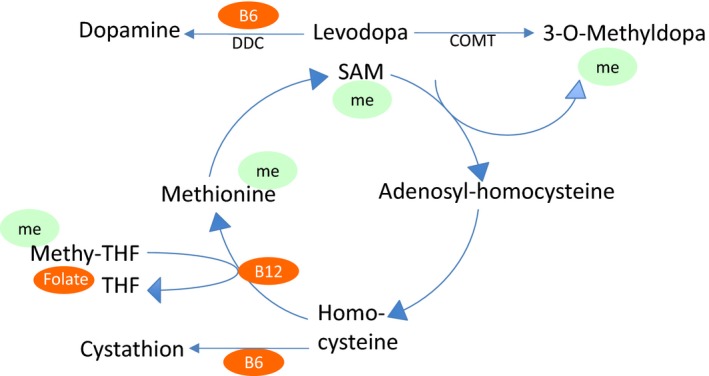
Levodopa metabolism within the plasma. B6 =  pyridoxine, B12 = cobalamin, COMT = catecholamine‐*O*‐methyl‐transferase, DDC = dopamine decarboxylase, me = methyl group transfer, Methyl‐THF = methyltetrahydrofolate, SAM = *S*‐adenosyl‐methionine

As neuropathy occurs in levodopa‐treated IPD, most researchers have concentrated on plasma changes in the substrates of the levodopa metabolism. Briefly, levodopa is metabolized via catecholamine‐*O*‐methyl‐transferase (COMT) to 3‐*O*‐methyldopa. This step requires a methyl group from *S*‐adenosyl‐methionine (SAM). The further metabolism leads to the formation of homocysteine, which is either remethylated to methionine in a cobalamin‐ (vitamin B12) and folate (vitamin B9)‐dependent reaction, or metabolized to cysteine. This latter step requires pyridoxine (vitamin B6) as a cofactor. Pyridoxine is also co‐factor in the decarboxylation of levodopa to dopamine (Figure 1).

Cobalamin deficiency as a potential cause of neuropathy in IPD has been investigated extensively, but with conflicting results: in levodopa‐treated IPD patients, cobalamin has been shown to be lower in those with neuropathy than in those without it (Ceravolo et al., 2013; Mancini et al., 2014); some researchers have found an association between cobalamin levels and levodopa doses (Rajabally & Martey, 2013; Toth et al., 2010), but this finding could not be confirmed by others (Mancini et al., 2014). Also folate deficiency (Rajabally & Martey, 2013) and levodopa itself, especially LCIG and its ingredients or its way of resorption (Jugel et al., 2013), have been suspected to be involved in the development of neuropathy in IPD.

Pyridoxine deficiency has rarely been studied systematically in this context, although case reports have described a possible association (Klostermann, Jugel, Muller, & Marzinzik, 2012; Urban et al., 2010). We too have observed dramatic changes of pyridoxine levels and just a mild decrease in cobalamin and folate in one of our patients in the first months after starting LCIG. This case was associated with subacute neuropathy (case 1, see below). This observation led us to assess the rest of our LCIG‐treated patients with respect to plasma levels of B vitamins, homocysteine, and neuropathy, and to compare their data to orally treated controls.

The objectives of this study were to determine whether the mode of levodopa application has an influence on plasma levels of pyridoxine, folate, cobalamin, and homocysteine as possible triggers of neuropathy. Next, we wanted to assess whether LCIG treatment is associated with a higher incidence of neuropathy than oral pharmacotherapy in patients with advanced IPD.

## 2 Materials and Methods

We searched our hospital database for data of advanced IPD patients treated either with LCIG or with oral dopaminergic drugs that had been hospitalized between January 2012 and December 2014 and for which plasma levels of pyridoxine (vitamin B6) were available. Vitamin levels were obtained irrespective of the cause of hospitalization, but only from those IPD patients that were taken care of by our movement disorders team.

For these identified patients, clinical data including gender, age, duration of IPD, Hoehn & Yahr stage (H&Y), UPDRS III in On condition, and current medication were collected. Of special interest were daily doses of levodopa (LDD); for other dopaminergic drugs, daily levodopa equivalent doses (LEDD) were calculated (Grosset, Needleman, Macphee, & Grosset, 2004; Tomlinson et al., 2010). In order to investigate whether both groups were comparable with regard to their dopaminergic demand, for LCIG patients current LDD/LEDD as well as the latest LDD/LEDD before starting LCIG treatment were obtained. All available plasma levels of folate, cobalamin, and homocysteine, and neurographies were collected. These plasma levels had originally been determined as a part of a routine laboratory testing in advanced IPD and therefore had been assessed independently of the presence of clinical or electrophysiological signs of neuropathy. Only patient data with results (clinical data, laboratory values and electrophysiology study results) obtained from the same time point were included into the analysis.

Only neurographies which included a complete exam with unilateral median, peroneal, tibial, and sural nerve were included, and were assessed with regard to motor and sensory conduction velocity, and amplitudes of the compound muscle action potential, or antidromic sensory potential, respectively. Neuropathy was defined as abnormal values for either conduction velocity or amplitudes in more than one nerve.

Statistical analyses were performed using GraphPad Prism 5.0 (GraphPad Software Inc, La Jolla, CA). Student′s *t* test and Mann–Whitney U tests were performed for clinical and demographic comparisons between the LCIG and orally treated patients. Significance was set at *p* < .05 for all analyses. Correlations between LDD/LEDD and vitamin plasma levels were assessed using Spearman′s correlation.

Analysis of this data was approved by the local ethics committee; all subjects gave informed consent to have their data analyzed anonymously in this study.

## 3 Results

In the period between 2012 and 2014, we identified 22 IPD patients treated with LCIG and 42 orally treated patients who had been admitted to our clinic. Thirteen LCIG patients had to be excluded because vitamin levels were unavailable, and one because LCIG therapy had been stopped 3 months before admission. Of those eight patients enrolled, six LCIG patients had been admitted because of technical complications of the PEG‐tube system, two patients because of subacute neuropathy. In 13 of the 42 orally treated patients, vitamin levels were available, and all of them had been admitted to optimize medical treatment of motor fluctuations, and none because of neuropathy or psychiatric complications. In the LCIG group, four patients had STN DBS; in one of them, it had been terminated due to intolerable side effects when LCIG was initiated. In the medical treatment group, two had ongoing STN DBS.

**Figure 2:**
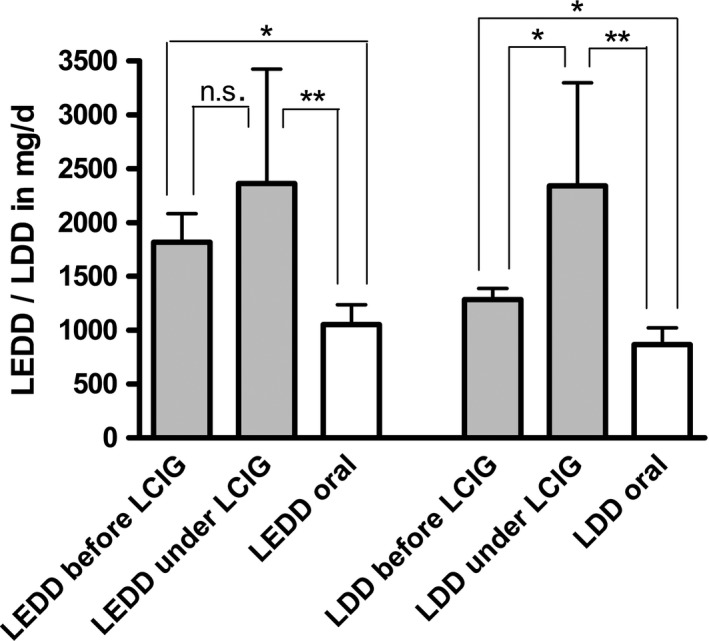
Dopaminergic treatment prior to and during levodopa/carbidopa intestinal gel LCIG treatment [mean + *SD*]. LEDD levodopa equivalent daily dose, LDD levodopa daily dose. *p < 0.04; **p < 0.004; n.s. = not significant

Both groups did not differ regarding gender, age, and motor impairment as measured by UPDRS III (on) and H&Y. Disease duration was slightly longer in the LCIG group, yet this difference did not reach significance (Table 1). Patients in the LCIG group had a significantly higher need for dopaminergic drugs even before the start of LCIG infusion therapy (Table 1, Figure 2, Mann–Whitney U test). Two patients were on 24/7 LCIG infusion. Their LDD was 2,429 ± 960, but not significantly higher than the LDD of the 16 hr infusion patients (1,917 ± 1,048).

**Table 1:** Epidemiological data and vitamin plasma levels

	LCIG *n* = 8	Oral *n* = 13	*p*‐value
Gender [m/f]	5/3	6/7	.55
Age [years]	69,8 ± 6,7	72,8 ± 6,8	.27
PD duration [years]	19,5 ± 6	13,7 ± 9,4	.06
Hoehn & Yahr	4 ± 1,3	4 ± 0,7	.63
UPDRS part III	21,8 ± 16,3	28,7 ± 13,9	.35
LDD [mg/day]	2,342 ± 956	865.8 ± 567	.0021
LEDD [mg/day]	2,360 ± 1,064	1,051 ± 662	.0034
LCIG duration [months]	37,4 ± 32,2	n/a	n/a
LDD increase with LCIG [mg/day]	963,1 ± 740,8	n/a	n/a
Pyridoxine [3.6–18 μg/L]	2.4 ± 1.4	5.5 ± 3.5	.01
Cobalamin [191–663 ng/L]	267.3 ± 85	321.4 ± 143.5	.48
Folate [4.6–18.7 μg/L]	7.8 ± 4.0	8.1 ± 3.0	1
Homocysteine [6.3–15.1 μmol/L]	33.7 ± 25	22.2 ± 6.9	.34

The change from oral treatment to LCIG had led to a further significant increase of LDD (before LCIG 1,286 ± 269 mg/day, during LCIG 2,342 ± 956 mg/day, *p* = .03). LEDD increased as well (before LCIG 1,814 ± 705 mg/day, during LCIG 2,360 ± 1,064 mg/day), however, this was not significant (two‐sided Wilcoxon test). As a result, LDD and LEDD were significantly higher in the LCIG group than in the orally treated group at the point of laboratory assessment (Figure 2, Mann–Whitney U test).

In six out of eight LCIG patients and 11 out of 13 orally treated patients, electrophysiology data was available. All patients in the LCIG group showed neuropathy: in fact, it occurred in virtually all examined peripheral nerves. With one exception, it was axonal sensory or sensorimotor neuropathy, that is, amplitudes of sensory or motor responses were either low or undetectable at all. In the oral treatment group, axonal sensory or sensorimotor neuropathy was found in eight of 11 patients, but compared to LCIG patients, it was generally milder with respect to the extent of axonal damage as well as to the number of affected nerves (Table 2).

**Table 2:** Electroneurographic data. Values in brackets are the cut‐off values established in our neurophysiology laboratory. Affected [%] denotes the percentage of examined nerves with either abnormal amplitude or abnormal velocity

			LCIG *n* = 6	Oral *n* = 11	*p*‐value
Median	Motor	Amplitude [>7.0 mV]	7.2 ± 1.2	6.8 ± 1.0	.83
		Velocity [>45 m/s]	47.6 ± 1.3	52.5 ± 1.7	.09
		Affected [%]	40	36	
	Sensory	Amplitude [>6.0 μV]	3.0 ± 1.8	6.8 ± 1.2	.09
		Velocity [>45 m/s]	26.2 ± 10.7	43.7 ± 4.6	.09
		Affected [%]	75	55	
Peroneal	Motor	Amplitude [>2.0 mV]	1.0 ± 0.50	2.4 ± 0.5	.10
		Velocity [>40 m/s]	34.3 ± 6.9	40.1 ± 4.3	.47
		Affected [%]	100	45	
Tibial	Motor	Amplitude [>4.0 mV]	1.5 ± 0.7	3.6 ± 1.0	.17
		Velocity [>40 m/s]	30.4 ± 1.8	39.8 ± 2.0	.009
		Affected [%]	100	67	
Sural	Sensory	Amplitude [>4.0 μV]	0.7 ± 0.7	2.6 ± 1.1	.30
		Velocity [>40 m/s]	11.6 ± 11.6	21.3 ± 7.5	.49
		Affected [%]	100	73	

**Figure 3:**
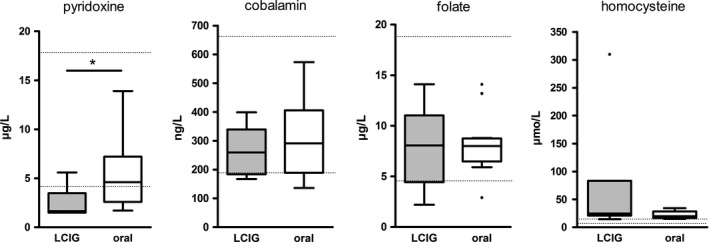
Serum levels of B vitamins and homocysteine. Dotted lines indicate normal ranges. Black dots mark outliers beyond 1.5‐fold the interquartile range. *p = .01

**Figure 4:**
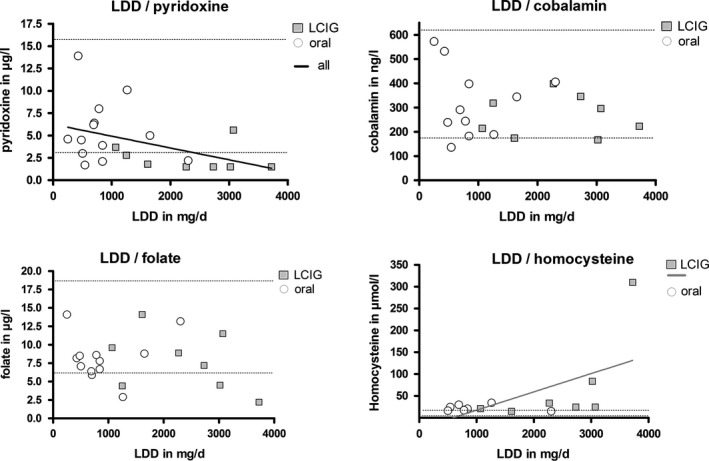
Correlations of plasma levels with levodopa daily dose (LDD). Dotted lines indicate normal range. Significant correlations have only been found for LDD and pyridoxine in general (r = −0.49, *p* = .02) and for LDD and homocysteine in levodopa/carbidopa intestinal gel LCIG patients (r = 0.78; *p* = .03). The bold lines show the results of the linear regression analysis for LDD and pyridoxine in general (*p* = .0577; R square = 0.18) and LDD and homocysteine in LCIG patients only (*p* = .02; R square = 0.36)

Of all plasma vitamin levels, pyridoxine was decreased most, and it was significantly lower in the LCIG group. All other parameters did not differ between the two groups: cobalamin and folate were within the lower reference range, and homocysteine highly elevated in both groups (Table 1, Figure 3). LDD correlated significantly with the decrease in pyridoxine levels (r = −0.49, *p* = .02) in all patients, and with the increase in homocysteine levels in the LCIG group (r = 0.78; *p* = .03), but with none of the other plasma levels (Spearman′s correlation coefficient, Figure 4).

The two cases of subacute axonal sensorimotor neuropathy in the LCIG group deserve special mentioning.

Case 1 was a 64‐year‐old female patient with a history of IPD for 14 years and severe motor fluctuations with a rapid switch between Off and heavy dyskinesia despite a combination of levodopa/carbidopa, entacapone, rotigotine, STN DBS, and apomorphine injections on a PRN basis. Before LCIG, she had an LDD of 1,450 mg and an LEDD of 2,990 mg. When LCIG was started, STN DBS was terminated because of intolerable side effects, and the dopamine agonists and COMT inhibition were discontinued resulting in a 16 hr LCIG monotherapy with an LDD of 3,860 mg. With this unusually high LDD, she was continuously in an On condition with no or mild dyskinesia, able to walk without a stroller at any time, and was very pleased with the result. Three months after LCIG had been started, she presented with a 2‐week history of sensory ataxia with astasia/abasia. Neurographies had revealed reduced amplitudes (i.e., axonal damage) before LCIG; however, 3 months later, no response whatsoever could be found anymore. Pyridoxine had dropped from 5.0 μg/L to below the threshold of detection (i.e., <1.5 μg/L), and homocysteine had shot up from 16.2 to 309.8 μmol/L. Cobalamin had dropped from 441.9 ng/L to 223 ng/L, and folate from >20 μg/L to 2.2 μg/L. LCIG was immediately stopped and replaced by subcutaneous apomorphine infusion; pyridoxine, cobalamin, and folate were substituted intravenously. However, the patient died from cerebral infarction one week after admission.

Case 2 was a 57‐year‐old male patient suffering from IPD for 14 years. His pre‐LCIG, LDD, and LEDD could not be obtained; however, 13 months after starting LCIG, he developed paresthesias in all limbs in a period of 4 weeks at an LDD of 3,108 mg. Neurographies revealed pronounced axonal sensory damage. Pyridoxine was <1.5 μg/L, homocysteine was 83.3 μmol/L, cobalamin 167 ng/L, and folate 4.5 μg/L. Due to lack of acceptable alternatives, LCIG was continued; i.v. substitution of pyridoxine, cobalamin, and folate resulted in a marked decrease of homocysteine to 32 μg/L after 1 week, and paresthesias remitted within months.

## 4 Discussion

The objectives of this study were to determine whether the application of levodopa either with oral or LCIG treatment has an influence on plasma levels of B vitamins and homocysteine in patients with advanced IPD, and if this is associated with a higher frequency of neuropathy in LCIG than oral pharmacotherapy.

The groups we compared were not significantly different with respect to age, gender, and H&Y; the mean disease duration in the LCIG group was 6 years longer, and LCIG patients scored better than orally treated patients on the UPDRS III, but both differences were statistically insignificant. The significant and most striking difference was that patients treated with LCIG had a more than 2.5‐fold higher dose of levodopa/carbidopa per day (LDD).

The higher LDD in the LCIG group was not unexpected, as LCIG allows switching from multiple dopaminergic drugs to levodopa monotherapy. However, also the LEDD was more than twofold higher than in the oral group demonstrating that LCIG patients required higher dopaminergic stimulation. When comparing pre‐LCIG LEDD to post‐LCIG LEDD, patients had 1.3‐fold higher dopaminergic demand during LCIG. We ascribed this higher dopaminergic demand in the LCIG group not to disease progression, but rather attributed this to the fact that some LCIG patients had 24/7 infusion, and in others subthalamic deep brain stimulation had been terminated because of intolerable stimulation‐induced side effects.

Neuropathy was found in 81% of patients (14 of 17) in whom electrophysiology data was available. It was predominantly axonal in nature. This proportion was much vaster than previously reported prevalences, which range from 5% to ~40% (Ceravolo et al., 2013; Jugel et al., 2013; Merola et al., 2014, 2016; Rajabally & Martey, 2011; Shahrizaila et al., 2013; Toth, Brown, Furtado, Suchowersky, & Zochodne, 2008; Toth et al., 2010). The frequency in our LCIG group was 100% (6 of 6), in the oral group 73% (8 of 11). Why is that so?

Levodopa therapy has been identified to be a risk factor for neuropathy (Mancini et al., 2014; Rajabally & Martey, 2013; Toth et al., 2008); however, dose dependency has not yet been studied systematically: one recent study found that IPD patients with neuropathy had an LDD of 1,180 mg, while those without neuropathy had an LDD of 775 mg (Mancini et al., 2014), and like in our study, there were no differences regarding age, gender, and H&Y stage. Our sample, however, had higher LDDs and longer disease durations and therefore very likely a higher exposure to levodopa. This might have contributed to the higher incidence of neuropathy we found. A possible dose dependency is also supported by our observation that patients with LCIG had more severe axonal loss and more nerves affected than the oral group. This finding is exactly the same described in another study examining neuropathy in LCIG treated in comparison to orally treated patients (Jugel et al., 2013).

The frequency of subacute neuropathy during LCIG has not been established yet. In the largest study so far, subacute neuropathy was found in four out of 50 patients (8%) (Mancini et al., 2014). In our clinic, two out of 22 patients receiving LCIG under regular surveillance between 2012 and 2014 had been admitted because of subacute neuropathy, resulting in a similar frequency of 9%. However, subacute neuropathy is not restricted to LCIG, but has been found in orally treated patients, too (Kimber, Blumbergs, & Thompson, 2013), interestingly in patients receiving LDD > 2000 mg/day.

What is the influence of B vitamins?

As shown in Figure 1, the metabolism of levodopa consumes cobalamin, folate, and pyridoxine which act as methyl groups donators; when these substrates are spent, homocysteine accumulates. Therefore, it seems obvious that with increasing doses of levodopa the levels of cobalamin, folate, and pyridoxine decrease, and levels of homocysteine rise. Looking at our results, this is exactly the case—cobalamin and folate were low in both groups, but still within normal range, homocysteine was high above upper limit. This result is in line with previous findings (Ceravolo et al., 2013; Mancini et al., 2014; Merola et al., 2014; Rajabally & Martey, 2011; Santos‐Garcia, Macias, Llaneza, Grande, & Fuente‐Fernandez, 2011; Toth et al., 2010). All three values did not significantly differ between LCIG and orally treated patients. Cobalamin and folate levels did not correlate with LDD, neither in LCIG nor in orally treated patients. In conclusion, we cannot rule out an influence of low folate and cobalamin levels on the development of neuropathy, but do not consider it as the main cause.

Pyridoxine, however, was low in both groups, but significantly lower in the LCIG group. When analyzed groupwise (i.e., LCIG and oral group separately), statistical analysis did not show a correlation of LDD with pyridoxine levels. Analysis of both groups as a whole, however, showed a significant inverse correlation between pyridoxine levels and LDD (*p* = .02). A closer look on single data revealed that with an LDD of 2,000 mg/day or higher, virtually all patients developed pyridoxine deficiency irrespective of the route of levodopa application. Again, this perfectly matches previous descriptions of neuropathy occurring at LDDs > 2,000 mg in oral patients (Kimber et al., 2013). Furthermore, we observed a significant correlation between LDD and homocysteine levels, although this was only true for the LCIG‐treated patients.

Why is it that of all B vitamins involved in the levodopa metabolism, pyridoxine is lowest in levodopa‐treated patients? Why is this probably a dose‐dependent phenomenon, and why is it so obvious in LCIG? It has been speculated that something in LCIG might be the cause; for example, the methylcellulose gel could hamper jejunal membrane functions (Jugel et al., 2013). We hypothesize that the reason might be the action of carbidopa in LCIG: Carbidopa is widely known as the decarboxylase inhibitor that inevitably comes along in LCIG and most oral levodopa formulations. Carbidopa irreversibly binds to and permanently deactivates free pyridoxine as well as pyridoxine‐dependent enzymes such as many decarboxylases; it thereby depletes the pyridoxine reserve pool (Cellini, Montioli, Oppici, & Voltattorni, 2012; Daidone et al., 2012). Thus, a high dose of levodopa/carbidopa—irrespective of the formulation—may be the cause of pyridoxine deficiency.

Pyridoxine is acting as a cofactor in a variety of metabolic reactions, and its deficiency itself is regarded a cause for neuropathy (Hammond, Wang, Dimachkie, & Barohn, 2013). This might be due to the antioxidant effects of pyridoxine (Shen, 2015). Additionally, pyridoxine deficiency increases homocysteine levels, and high doses of pyridoxine have been shown to reduce homocysteine levels (Miodownik, Lerner, Vishne, Sela, & Levine, 2007). Hyperhomocysteinemia is a common finding in levodopa therapy (Antonini, Bondiolotti, Natuzzi, & Bareggi, 2010; Mancini et al., 2014; Miller et al., 2003), and has been suggested to be a cause of neuropathy via a neurotoxic effect (Merola et al., 2016; Muller, 2008; Muller et al., 2013), as it is associated with reduced sensory nerve amplitudes (Muller, Renger, & Kuhn, 2004). We cannot rule out that cobalamin and folate play a role in the genesis of neuropathy, but our data strongly indicate that pyridoxine deficiency is a major factor.

It remains uncertain if slowly progressive neuropathy and subacute neuropathy observed under LCIG are identical or different in nature. In the documented cases of subacute neuropathy, cobalamin was usually in low‐normal range (Klostermann et al., 2012; Urban et al., 2010) or reduced (Galazky et al., 2014). Pyridoxine levels were only sporadically measured; usually markedly reduced levels were found (Galazky et al., 2014; Klostermann et al., 2012; Mancini et al., 2014; Urban et al., 2010). A universal explanation for subacute neuropathy has not been given so far. In our series, the subacute neuropathy cases had particularly high levels of homocysteine and low pyridoxine, although low pyridoxine values were found in cases with slowly progressive neuropathy as well. The rapid increase in levodopa/carbidopa, as it is usually the case when initiating LCIG therapy, may be an explanation. This would be supported by the observation made in case 1, in which an increase in levodopa/carbidopa by factor 2.6 dramatically decreased pyridoxine and increased homocysteine. Clinically, the patient presented with subacute neuropathy after 3 months, a scenario that has been described elsewhere as well (Klostermann et al., 2012).

Our study unfortunately has a couple of limitations, the retrospective character with no randomization, not all data available for all patients, and the small sample size being the most important ones. Moreover, we analyzed only data of patients in whom pyridoxine levels were available, so not every IPD in‐patient was investigated. Even though pyridoxine levels and neurographies were determined on a routine basis and independently of clinical signs of neuropathy, we cannot rule out a bias due to different causes of hospitalization. Furthermore, we do not have standardized clinical data on neuropathic symptoms, let alone on their severity, and due to the retrospective character of this study we cannot rule out neuropathy of other origins in one or the other case. Finally, the validity of our results is limited by the fact that the LCIG group showed longer disease duration and higher dopaminergic demand. Thus, we cannot exclude that the differences we found are—at least partly—due to IPD itself or different durations of exposure to levodopa/carbidopa.

In summary, our study shows that neuropathy is very common in advanced IPD patients treated with high doses of levodopa/carbidopa irrespective of the formulation. Our results show an association of low pyridoxine levels with high LDDs in a dose‐dependent manner. We deem pyridoxine deficiency induced by metabolizing levodopa as well as induced by irreversible binding of pyridoxine to carbidopa to be two of the major mechanisms. The risk may be extremely high in patients receiving LDDs beyond 2,000 mg, and in those, in which LDD is rapidly increased within a short period of time, as rather often is the case during the initiation of LCIG therapy. We therefore recommend routine assessments of neurographies, pyridoxine, folate, and cobalamin levels in any advanced IPD patients having high LDD in general, and especially before and after initiating LCIG. We furthermore suggest to consider supplementation when severe shortage of pyridoxine is found. In these cases, ongoing monitoring of pyridoxine levels, neuropathic features, and the worsening of parkinsonian symptoms is essential, because high doses of pyridoxine themselves can cause neuropathy.

Future studies are needed to address a couple of urgent questions on this important topic: the most important topic certainly is to establish a clear relationship between pyridoxine levels and neuropathy on the ground of clinical and electrophysiological data, at best in a prospective study design. Furthermore, gathering information on dosage and duration of pyridoxine supplementation is fundamental.

## Financial Disclosures/Conflict of Interest

SL has received travel grants from Abbvie Deutschland. EC and AK have nothing to disclose. DD has nothing to disclose in relation to this study. CS is consultant to Abbvie Deutschland and has received speaker honoraria and travel grants from Abbvie Deutschland.

## Author Contribution

SL organized and executed the research project, executed the statistical analysis, wrote the first draft of manuscript, and critically reviewed manuscript. EC organized and executed the research project, critically reviewed the statistical analysis and the manuscript. AK executed the research project, designed and executed the statistical analysis, and critically reviewed the manuscript. DD executed the research project, critically reviewed the statistical analysis, and critically reviewed the manuscript. CS conceptualized, organized, and executed the research project, designed and critically reviewed the statistical analysis, and also reviewed the manuscript.
